# Energy Harvesting by Subcutaneous Solar Cells: A Long-Term Study on Achievable Energy Output

**DOI:** 10.1007/s10439-016-1774-4

**Published:** 2017-01-03

**Authors:** L. Bereuter, S. Williner, F. Pianezzi, B. Bissig, S. Buecheler, J. Burger, R. Vogel, A. Zurbuchen, A. Haeberlin

**Affiliations:** 1 Department of Cardiology, Bern University Hospital, 3010 Bern, Switzerland; 2 ARTORG Center for Biomedical Engineering, University of Bern, 3008 Bern, Switzerland; 3 Department of Cardiology, Solothurner Spitäler AG, 4500 Solothurn, Switzerland; 4 Laboratory for Thin Films and Photovoltaics, Swiss Federal Laboratories for Materials Science and Technology Empa, 8600 Duebendorf, Switzerland; 5 Haute Ecole Arc Ingénierie - University of Applied Sciences and Arts Western Switzerland (HES-SO), 2300 La Chaux-de-Fonds, Switzerland; 6 Department of Cardiology, Bern University Hospital and University of Bern, c/o ARTORG Center for Biomedical Engineering Research, Murtenstrasse 50, 3008 Bern, Switzerland

**Keywords:** Medical implants, Pacemaker, Photovoltaic, Light transmittance, Skin, Light exposure, Power, Feasibility

## Abstract

Active electronic implants are powered by primary batteries, which induces the necessity of implant replacement after battery depletion. This causes repeated interventions in a patients’ life, which bears the risk of complications and is costly. By using energy harvesting devices to power the implant, device replacements may be avoided and the device size may be reduced dramatically. Recently, several groups presented prototypes of implants powered by subcutaneous solar cells. However, data about the expected real-life power output of subcutaneously implanted solar cells was lacking so far. In this study, we report the first real-life validation data of energy harvesting by subcutaneous solar cells. Portable light measurement devices that feature solar cells (cell area = 3.6 cm^2^) and continuously measure a subcutaneous solar cell’s output power were built. The measurement devices were worn by volunteers in their daily routine in summer, autumn and winter. In addition to the measured output power, influences such as season, weather and human activity were analyzed. The obtained mean power over the whole study period was 67 *µ*W (=19 *µ*W cm^−2^), which is sufficient to power e.g. a cardiac pacemaker.

## Introduction

Electronic implants are usually battery powered, rarely with a rechargeable battery—which requires repeated recharging—or with a primary battery, which requires an implant replacement when the battery is depleted. In fact, implant replacements due to battery depletion are common and account for approximately 25% of implantations of cardiac pacemakers, which represent the majority of electronic implants.^12^ These re-interventions cause costs and expose the patient to a risk of complications. Moreover, it may be a stressful intervention for the patient. Finally, the size of an electronic implant is mainly governed by the battery volume, i.e. it could be designed smaller if not equipped with primary batteries.

As a promising alternative energy source, ambient sunlight could be used. Sunlight is a reliable and omnipresent energy source and a fraction of the ambient light penetrates the human skin. In particular, near-infrared light features good skin penetration.^1^ Thus, an implant is irradiated, yet implanted under the skin and the penetrating light could be converted into electrical energy by solar cells.

Recently, subcutaneously implantable pacemakers as well as sensors powered by solar cells have been proposed to overcome the battery-related limitations of contemporary devices. The working principle of such devices has been proposed by several groups from a technical and biological point of view.^3^,^6^,^7^,^15^,^16^ However, precise knowledge of the actual light exposure and expectable power output of such an implant in everyday life is lacking so far.

To investigate the real-life feasibility of an implant powered by solar cells, a wearable measurement device was developed that gathers the output power of solar cells suitable for such implants. A long-term validation study with 32 volunteers was performed for the duration of six months in Central Europe (Switzerland). The volunteers wore the measurement devices in their daily routine to determine the generated power as well as the influence of other factors such as weather or human behavior.

## Materials and Methods

### Measurement Device

A wearable measurement device that features solar cells was built. The primary task of this measurement device is to continuously monitor the solar cells’ output power. A key element is that the solar cells are covered by optical filters to simulate subcutaneously implanted solar cells.

#### Principle

**Figure 1:**
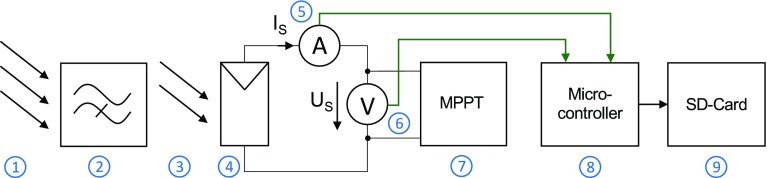
Block diagram of the measurement device showing the ambient light (*1*), which is attenuated by optical filters (*2*) that mimic the optical properties of human skin. The attenuated light (*3*) irradiates the solar cells (*4*), which are connected to a maximum power point tracker (*7*). The solar cells’ output power is monitored by a current- and voltage measurement circuit (*5*, *6*). A microcontroller (*8*) controls the measurement and stores the data onto a memory card (*9*).

The measurement device includes a circuitry to measure the solar cell’s power output and stores the data on a memory card. Figure 1 shows the block diagram of the measurement device: the ambient light (1) is filtered by dedicated optical filters (2) that exhibit similar optical properties as human skin. The filtered light (3) irradiates the solar cells (4), which are connected to a maximum power point tracker (MPPT, 7). The MPPT maximizes the power output of the solar cells, which is measured by current $I_{\text{S}}$ [A] (5) and voltage $U_{\text{S}}$ [V] (6). A microcontroller (8) controls the analog-to-digital converters (ADCs) from the power measurement (5, 6) and stores the data onto a memory card (9).

#### Optical Filters

To estimate the power output of a subcutaneous solar cell, the solar cells of the measurement device have to be covered by a material that exhibits similar optical properties as human skin. Real non-vital skin would change its optical and biologicals properties rapidly over time and would not allow reliable and reproducible results. Thus, an alternative material with stable properties had to be implemented. Optical properties of human skin are documented well in literature, *in vivo* 18 and *in vitro* 1,9,14,16,17 data of human skin are presented, giving the wavelength-dependent absorption coefficient $\mu_{\alpha }$ and transport scattering coefficient $\mu '_{\text{s}}$. However, reported data differs, especially in the near infrared range (NIR).^1^ Moreover, for this application, the major interest is the wavelength-dependent total light transmission ratio (i.e. transmittance = $T_{\lambda }$[%]).

To attain more data of the transmittance and to be able to also investigate skin with different layer thicknesses (dermis, subcutis), $T_{\lambda }$ of several ex vivo domestic pig skin flaps was measured, since anatomical and optical properties of pig skin are similar to human skin.^19^ In total, 16 skin samples (purchased from the slaughterhouse, size 15 × 15 mm) were cut out at different hairless sites. The total thickness of the samples (epidermis + dermis + subcutis) varied from 1.6 mm up to 6.8 mm. The skin samples were placed on a glass plate and an optically opaque frame with a translucent window of 4.5 × 4.5 mm was placed on the epidermis. Finally, $T_{\lambda }$ was measured with a spectrophotometer (UV-3600 UV–Vis-NIR Spectrophotometer, Shimadzu, Japan) over the wavelength range of 300 to 1500 nm in 2 nm steps. The measurement was done directly after purchasing the fresh skin and therefore, no significant changes of the optical properties are expected.^5^

Subsequently, the applicability of the transmittance results to skin-covered solar cells was investigated: The computed short circuit current per solar cell area $I_{{{\text{SC}}\_{\text{C}}}}$ [A m^−2^], calculated based on the transmittance results, and the measured short circuit current $I_{{{\text{SC}}\_{\text{M}}}}$ [A] were compared.

First, $I_{{{\text{SC}}\_{\text{C}}}}$ for a skin-covered, irradiated reference solar cell with known external quantum efficiency (${\text{EQE}}$ [%], ratio of incident photons to converted electrons), was calculated. $I_{{{\text{SC}}\_{\text{C}}}}$ was obtained by integrating the photon flux $\phi_{{{\text{AM}}1.5{\text{G}}}}$ [# photons m^−2^ s^−1^], in our case for the spectrum AM1.5G (100 mW cm^−2^), the previously measured transmittance spectra $T_{\lambda } (\lambda )$ and ${\text{EQE}}(\lambda )$ and multiplying the result with the electron charge $q$ = 1.6 × 10^−19^ C.

1 $$I_{{{\text{SC}}\_{\text{C}}}} = q \times \int {\phi_{{{\text{AM}}1.5{\text{G}}}} \left( \lambda \right)T_{\lambda } \left( \lambda \right){\text{EQE}}\left( \lambda \right){\text{d}}\lambda }$$

Second, the same skin samples were put on the standard reference solar cell (4.5 × 4.5 mm). On top of the skin samples, an optically opaque frame (10 × 10 mm) was stacked to prevent lateral irradiation. The setup was irradiated by a solar simulator (LS0811, LOT-Quantum Design, Germany) simulating 1 sun (100 mW cm^−2^, AM1.5G). The measured short circuit current $I_{{{\text{SC}}\_{\text{M}}}}$ was determined with a sourcemeter with four terminal sensing (Keithley 2400, Keithley Instruments, USA) during irradiation. The difference between $I_{{{\text{SC}}\_{\text{C}}}}$ and $I_{{{\text{SC}}\_{\text{M}}}}$ will be discussed in the Discussion section.

**Figure 2:**
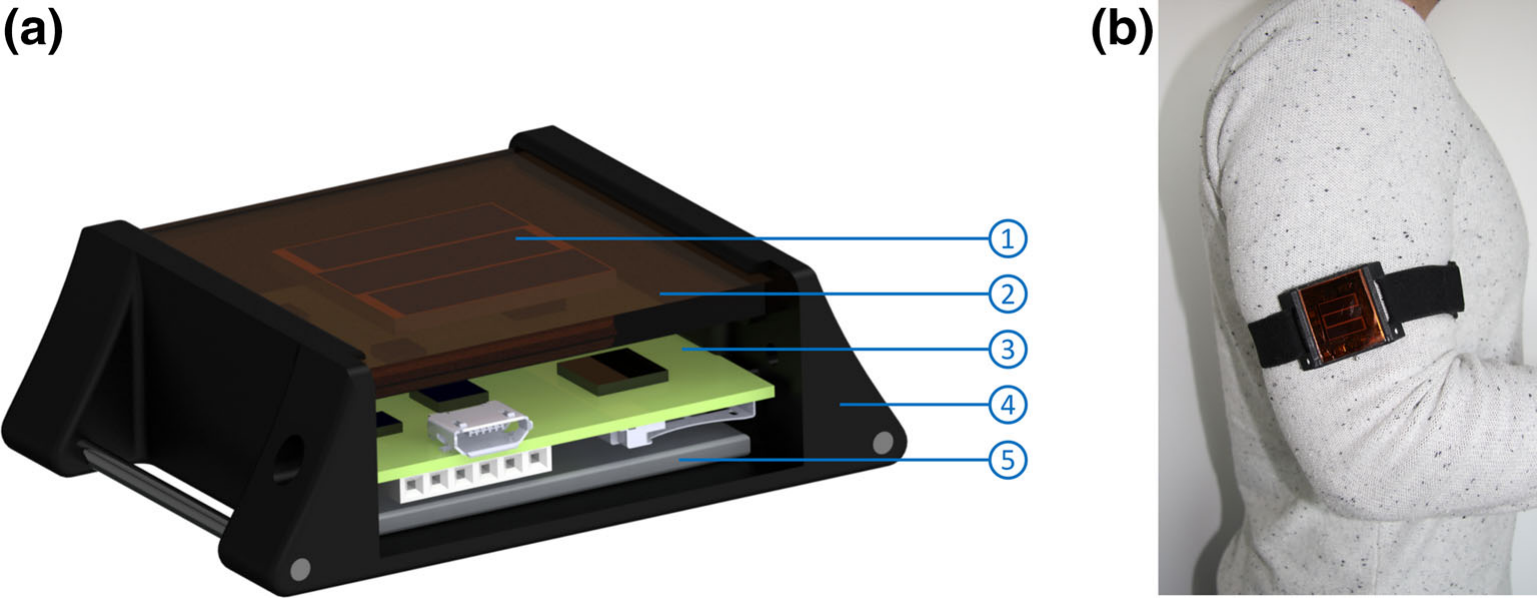
(**a**) Cross-sectional view of the measurement device. The solar cells (*1*) are located directly below the optical filters (*2*). Furthermore, the PCB (*3*) and battery (*5*) are enclosed in the housing (*4*). (**b**) Measurement device fixated on the upper arm.

**Figure 3:**
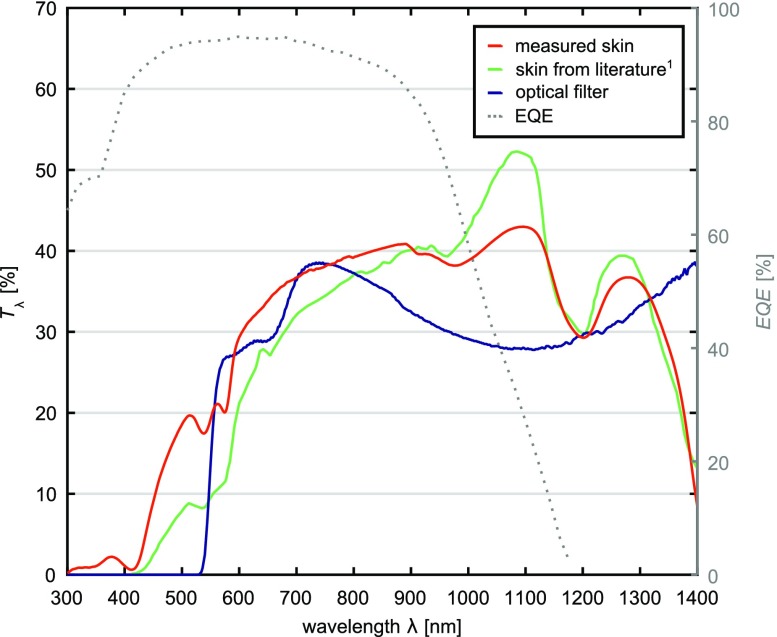
Transmittance spectra $T_{\lambda } (\lambda )$ of 2.3 mm thick skin and the optical filters (blue). Measured porcine skin sample (red), human skin according to Bashkatov *et al*.^1^ (green) and $EQE(\lambda )$ of the solar cells (dotted grey).

Based on the obtained transmittance results, a combination of two optical filters that emulate the transmittance profile $T_{\lambda } (\lambda )$ of 2.3 mm thick skin (Fig. 3) was determined: A 550 nm longpass (FGL550S, Thorlabs, Germany) and an absorptive neutral density filter (NE205B, OD: 0.5, Thorlabs, Germany) were stacked on top of each other. These filters are placed directly on top of the solar cells of the measurement device (Fig. 2). The wavelength-dependent short-circuit current $I_{\text{SC}} \left( \lambda \right)$ [A m^−2^] was computed to determine the performance of the optical filters compared to real skin. For full sun irradiation (AM1.5G, 100 mW cm^−2^), $I_{\text{SC}} \left( \lambda \right)$ is obtained by the following formula, whereas the transmittance spectra of either the optical filters or the 2.3 mm thick skin is used for $T_{\lambda } \left( \lambda \right)$.

2 $$I_{\text{SC}} \left( \lambda \right) = \phi_{{{\text{AM}}1.5{\text{G}}}} \left( \lambda \right)T_{\lambda } \left( \lambda \right){\text{EQE}}\left( \lambda \right)q$$

#### Solar Cells

The employed solar cells are monocrystalline silicon cells (KXOB22-12X1L, IXYS Corporation, USA) with an efficiency of 22%. Three cells are connected in series and the resulting total active area is 3.6 cm^2^. The three solar cells are aligned in parallel which results in a total outer dimension of 21 × 22 mm.

#### Measurement Circuit

The maximum power point tracker (BQ25570, Texas Instruments, USA) modulates its input impedance to regulate $U_{\text{S}}$ at 80% of the solar cells’ open-circuit voltage ($U_{\text{OC}}$). At $U_{\text{S}}$ = 80% $U_{\text{OC}}$, the solar cells exhibit maximum output power. The MPPT obtains the latest value for $U_{\text{OC}}$ every 16 s, which takes 256 ms and during this time the solar cells' output current $I_{\text{S}}$ is zero. $I_{\text{S}}$ is measured by a high-side current-sense circuitry featuring a 1 Ω sense resistor connected in series between the solar cells and the MPPT. The voltage drop over the sense resistor is amplified (ADA4051, Analog Devices, USA) and digitized by a two-channel 16-Bit ADC (ADS1118, Texas Instruments, USA). The system makes use of the ADC’s internal programmable gain amplifier (PGA) to increase the resolution for currents in the *µ*A-range. The circuit can measure currents up to 15 mA with a dynamic resolution between 80 nA and 0.6 *µ*A, theoretically. This range covers full sunlight irradiation (AM1.5G, 100 mW cm^−2^) considering attenuation of the filters as described previously. The solar cell’s output voltage $U_{\text{S}}$ is buffered by a unity gain amplifier to minimize measurement induced error and then digitized with a resolution of 0.6 *µ*V by the same ADC, range $U_{\text{S}}$ = 0–2 V. The microcontroller coordinates the measurement: $I_{\text{S}}$ and $U_{\text{S}}$ are measured with a sampling frequency of 1 Hz. Finally, the values (date, time (h:min:s), $I_{\text{S}}$, $U_{\text{S}}$, PGA) are stored on a memory card. The electronic components are assembled on a printed circuit board (PCB). The device features a rechargeable battery that enables a continuous measurement for more than two weeks.

To increase the measurement device’s accuracy, individual static errors were determined: The error caused by the input offset voltage of the operational amplifiers as well as the gain error due to resistor tolerances were measured. These data were used to individually correct the data gained from the device measurement results. Finally, the accuracy was specified over the whole measurement range ($I_{\text{S}}$ = 0–15 mA, $U_{\text{S}}$ = 0–2 V) using a reference measurement device (Agilent N5705B/N6781A, Agilent Technologies, USA).

#### Housing

The individual parts of the measurement device are assembled in a 3D-printed housing (outer dimensions: 63 × 46 × 19 mm) that features an elastic cuff for fixation on the upper arm (Fig. 2b). This fixation location results in a similar declination angle of incident light as if the solar cells were implanted in the neck region (as intended to do for a cardiac pacemaker^7^,^15^). Figure 2 shows a cross-sectional view of the measurement device: The solar cells (1) are covered by the optical filters (2) that mimic the optical behavior of skin. To avoid shadowing of the housing frame, the solar cells are assembled directly below the filters. The PCB (3), including programming interface, memory card and charging interface as well as the battery (5) are accessible by a removable flap. Clefts are sealed with silicon which makes the device splash-proof.

### Study

#### Design

To validate the feasibility of subcutaneous solar energy harvesting, a long-term validation study with volunteers was performed for the duration of six months. The study participants were recruited via word of mouth. Every study participant wore the measurement device during a period of one week per season in summer (June–August), autumn (September–October) and winter (November–December). The date when the study participant had to wear the device was randomly assigned. The study participants were advised to wear the measurement device on the upper arm during the whole day, from morning to bedtime. In general, the volunteers were instructed to wear the device uncovered (i.e. over the clothes). However, the participants were instructed to cover the measurement device when wearing a neck covering (e.g. scarf, jacket with high collar), in order to simulate a neck implant. Predominant weather and activity was registered using a daily questionnaire (weather: sunny, partly sunny, cloudy, rainy/activity: working indoor, leisure time indoor, working outdoor, leisure time outdoor). Furthermore, discrimination between the age groups of 18–64 years and 65–89 years (retired persons) was made. All data were registered anonymously and no health-related data were recorded. Therefore, this study is not defined as a clinical trial by Swiss law.

#### Data Analysis

The main result of interest is the output power of the solar cells $P_{\text{S}}$ [W], which is calculated by multiplying the corresponding values of current $I_{\text{S}}$ and voltage $U_{\text{S}}$. This gives a power-profile for the whole day. $\overline{{P_{\text{D}} }}$ [W] is the arithmetic mean of one day (24 h) and $\overline{{P_{\text{M}} }}$ [W] is the arithmetic mean of a month (i.e. the mean of all $\overline{{P_{\text{D}} }}$ during one month). The recordings usually cover 24 h of a day. In rare cases, study participants forgot to wear the logger and reported this in the questionnaire as a lack of data. In this case, data was included in the analysis if at least 12 h were valid (00:00–12:00 or 12:00–24:00), otherwise data was discarded. Calculation and statistical analysis was performed with MATLAB R2015b (Mathworks, USA). The output power for statistical considerations and $\overline{{P_{\text{M}} }}$ are reported as mean values with standard deviation. A two-sided Wilcoxon signed-rank test was performed for statistical analysis. A *p* value ≤0.05 was considered significant.

## Results

In the first part of the following section, measurement device specific results are presented. In particular, the performance of the optical filters compared to real skin is shown. In the second part, the study results are presented and statistically analyzed.

### Measurement Device

#### Optical Filter

The fact that porcine skin exhibits similar properties as human skin was also observed in the measurements:

Figure 3 shows the measured transmittance $T_{\lambda } (\lambda )$ for a 2.3 mm thick skin sample (red). For comparison, $T_{\lambda } (\lambda )$ of human skin^1^ with the same thickness is shown (Fig. 3, green). The transmittance $T_{\lambda } (\lambda )$ of the optical filter combination is shown in blue (Fig. 3). Only transmittance values at wavelengths in the range of 300–1200 nm are relevant, since beyond this range, the solar cell’s external quantum efficiency (${\text{EQE}}$) is zero and no energy can be gathered. At lower wavelengths ($\lambda$ < 400 nm), the light is completely blocked by the skin. Between the wavelengths 400 and 600 nm, a strong increase in transmittance is obtained. In particular, in the NIR ($\lambda$ > 750 nm), the solar cells still exhibit considerable external quantum efficiency (${\text{EQE}}(\lambda )$, dotted grey line).

**Figure 4:**
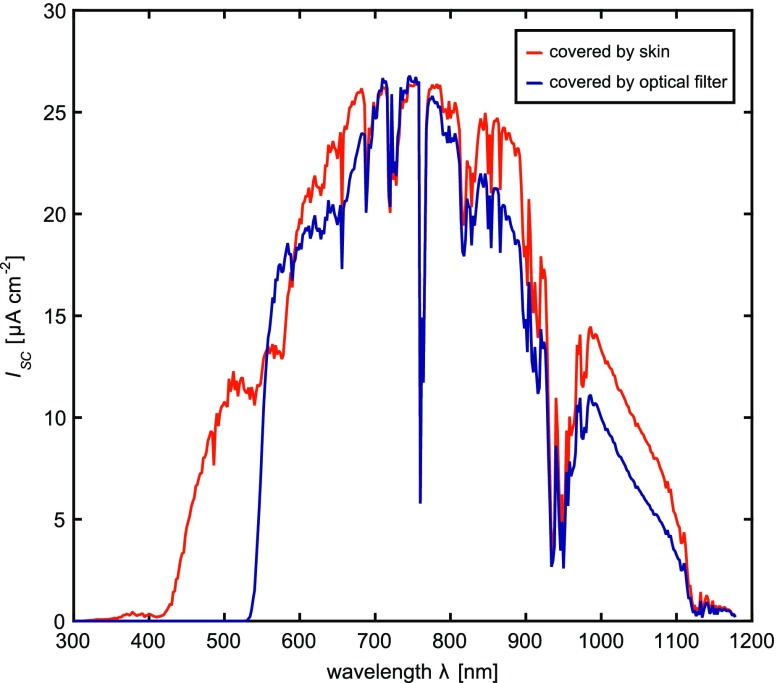
Comparison of the wavelength-dependent short-circuit current $I_{\text{SC}} (\lambda )$ when the solar cells are covered with 2.3 mm skin (red) or with optical filters (blue) and irradiated with 1 sun (AM1.5G).

The resulting wavelength-dependent short circuit current $I_{\text{SC}} (\lambda )$ of the solar cells covered by the filters as well as the skin was assessed to evaluate over- or underestimation of the power measured with the portable devices on the field: Fig. 4 compares $I_{\text{SC}} (\lambda )$ for solar cells covered by 2.3 mm thick skin (red) and by the optical filter combination (blue), respectively, irradiated by 1 sun (AM1.5G, 100 mW cm^−2^). The resulting profile of $I_{\text{SC}} (\lambda )$ with the filters shows good accordance in the relevant spectral band. The short circuit current $I_{{{\text{SC}}\_{\text{C}}}}$ when covered by the filters is 19% lower than when covered by the measured skin and 6% lower compared to the skin from literature.^1^

#### Measurement Circuit

In total, 10 devices were built. The measured values were individually corrected for the device-specific offset voltage and gain error during offline processing. With the correction, the device’s accuracy can be specified as followed: $I_{\text{S}}$ [0–10 *µ*A: 1% + 0.1 *µ*A, 10–100 *µ*A: 1% + 1 *µ*A, 100 *µ*A–1 mA: 1% + 4 *µ*A, 1–15 mA: 1% + 20 *µ*A] and $U_{\text{S}}$ [0–2 V: 0.2% + 2 mV].

### Study Results

The study included 32 participants (13 female, 19 male) in two age groups (younger than 65 years: 24 participants, 65 years or older: eight participants). The measurements started on June 21st, 2015 and lasted until the end of the year 2015. In total, 672 days of data were gathered, of which 10% were excluded from analysis based on the previously described criteria, which results in 608 days of data for analysis. All measurements were performed in Switzerland (latitude: 46°–47.5°, longitude: 6°–10°).

**Figure 5:**
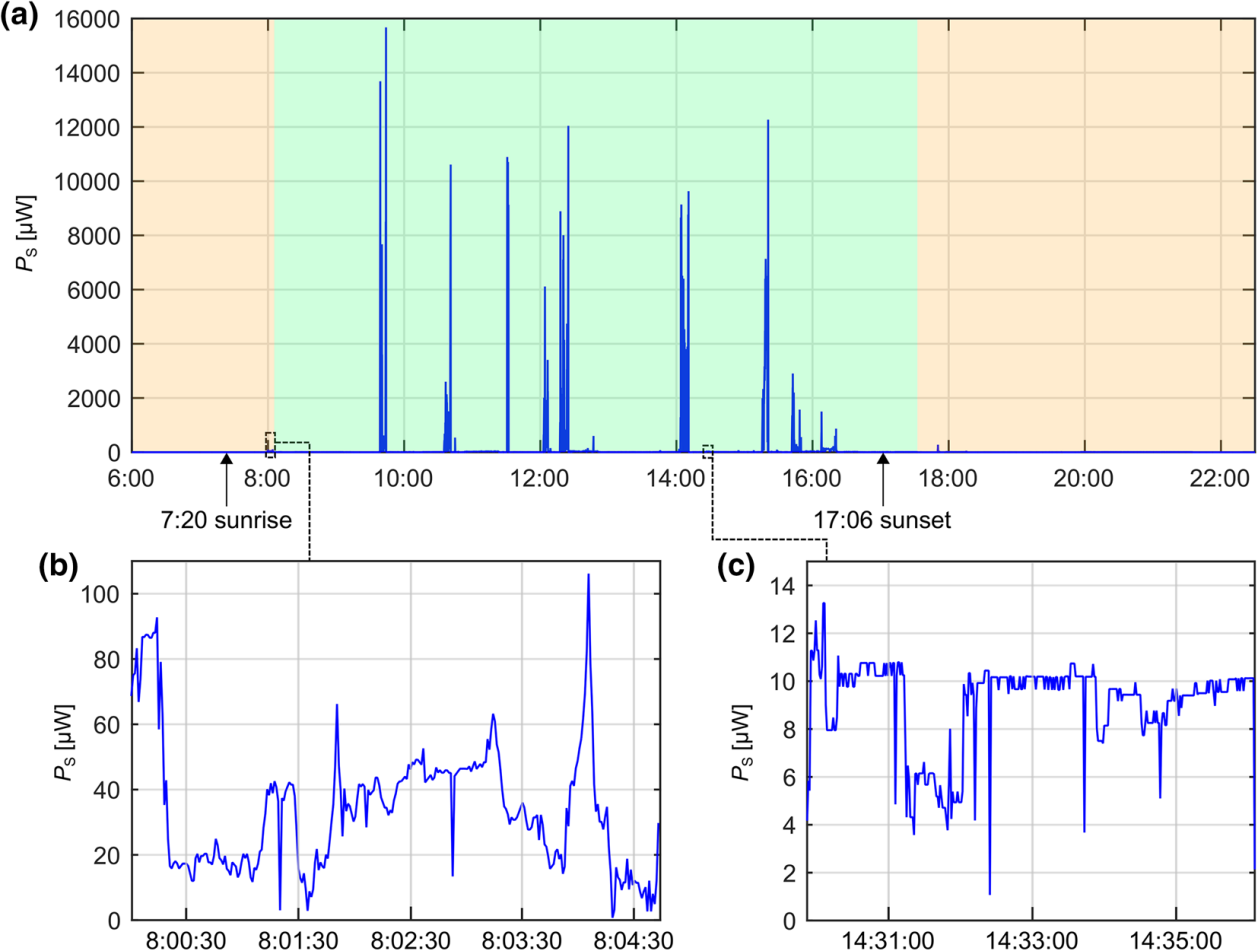
Example of the measured power $P_{\text{S}}$ on November 6th, 2015 (winter). The weather was predominantly sunny and the participant was working indoors. (**a**) $P_{\text{S}}$ during the day (blue). The green and orange shaded areas represent the time when the participant was at work or at home, respectively. The power peaks indicate when the participant was outdoors. The mean power $\overline{{P_{\text{D}} }}$ on this day (24 h) was 51 *µ*W. (**b**) Zoom of $P_{\text{S}}$ on the way to work (outdoors), short after sunrise. (**c**) Zoom of $P_{\text{S}}$ while working in the office.

Figure 5 shows an example of the measured output power ($P_{\text{S}}$) during one day. The periods when the participant was outdoors are clearly discernible and are indicated by the power peaks (Fig. 5a), whereas $P_{\text{S}}$ was lower when the subject was indoors (Fig. 5c). Even short after sunrise, a considerable amount of power was measured outdoors (Fig. 5b, on the way to work).

**Figure 6:**
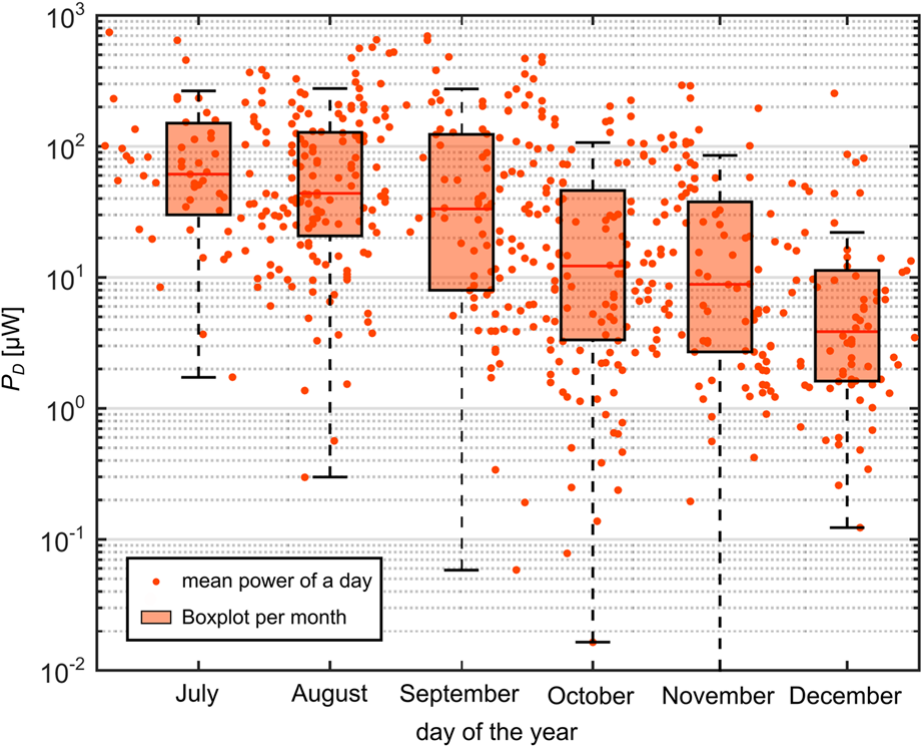
Mean power $\overline{{P_{\text{D}} }}$ of every measurement day (orange dots, from June 21st–December 31st) and the monthly mean $\overline{{P_{\text{M}} }}$ from July to December (boxplots without outliers).

Figure 6 shows the mean power $\overline{{P_{\text{D}} }}$ (orange dots) of every measurement day (data of all participants, in total 608 days). The monthly mean power $\overline{{P_{\text{M}} }}$ is shown as boxplots. A high variability of $\overline{{P_{\text{D}} }}$ was observed, ranging from <1 *µ*W up to 744 *µ*W. Towards winter, the monthly mean $\overline{{P_{\text{M}} }}$ decreases: July: 106 ± 123 *µ*W, August: 99 ± 123 *µ*W, September: 84 ± 130 *µ*W, October: 45 ± 79 *µ*W, November: 34 ± 56 *µ*W, December: 14 ± 34 *µ*W. The mean power for each season was 106 ± 130 *µ*W for summer, 66 ± 111 *µ*W for autumn and 27 ± 49 *µ*W for winter.

**Figure 7:**
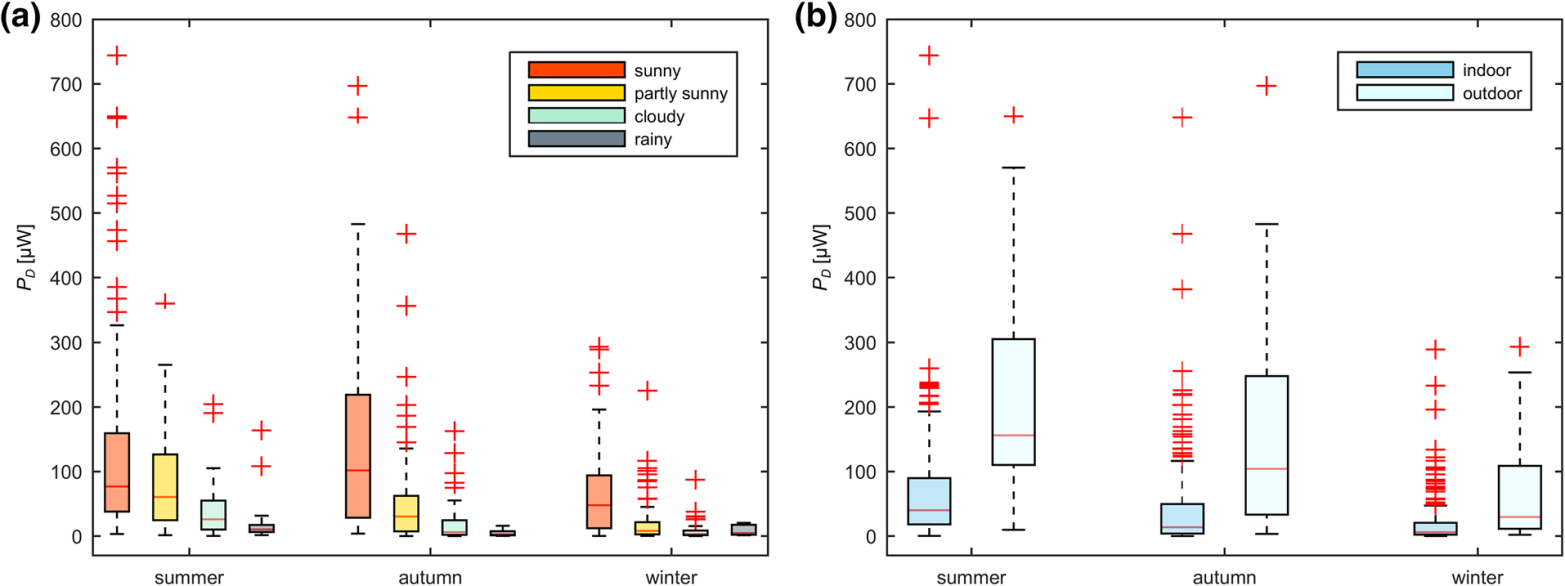
Seasonal distribution of the mean power $\overline{{P_{D} }}$ for different weather situations and activities. The graphs include the results of every participant (608 days). (**a**) Boxplot of the measured power dependent on the weather. (**b**) Boxplot of the measured power dependent on the activity (predominantly indoor/outdoor).

Figure 7a shows a boxplot of the distribution of the mean power $\overline{{P_{\text{D}} }}$ for the different seasons and the dominant weather. The total dominant weather was distributed as follows: 209 days sunny, 195 days partly sunny, 154 days cloudy and 50 days rainy. In general, $\overline{{P_{\text{D}} }}$ was higher for sunny weather and decreases towards cloudy and rainy weather. A large increase in $\overline{{P_{\text{D}} }}$ was observed when the subject was mainly outdoors, compared to indoor activities (Fig. 7b). However, the power increases for sunny weather, even when the participant remained indoors. For indoor periods, no difference between work or leisure related activities was obtained (*p* = 0.81). $\overline{{P_{\text{D}} }}$ also shows a high weather-dependent variability for outdoor activities. Overall, the mean power $\overline{{P_{\text{D}} }}$ indoor was 45 ± 77 *µ*W compared to 182 ± 164 *µ*W outdoor (*p* < 0.001). The mean power for all participants younger than 65 years was 61 ± 105 and 86 ± 117 *µ*W for the group above 65 years (*p* = 0.005). There was no difference in power output between genders (*p* = 0.16).

## Discussion

The obtained overall mean power is 67 ± 108 *µ*W (=19 ± 30 *µ*W cm^−2^), which is enough to completely power e.g. a pacemaker or at least extend the lifespan of any other active implant. In the first part of the discussion, measurement device specific aspects are discussed. In the second part, the study data are interpreted and discussed.

### Measurement Device

The MPPT regulates the solar cells' output voltage $U_{\text{S}}$ at 80% of $U_{\text{OC}}$, which was measured every 16 s. However, the analysis of the time-dependent measured power $P_{\text{S}}$ showed a very dynamic behavior of the incident irradiation on the solar cells (Fig. 5b, e.g. from the movement of the person). By measuring $U_{\text{OC}}$ at a higher sampling frequency, the solar cell’s dynamic operating point would be optimized and the overall power increased.

The optical filters were used to mimic the optical properties of skin but do not perfectly reproduce the transmittance spectra of real skin. The solar cell’s wavelength-dependent short circuit current $I_{\text{SC}} (\lambda )$ when covered by the optical filters or by skin (Fig. 4) showed that the filters completely cut light at wavelengths smaller than 550 nm, whereas skin still transmits a part of the light in this range. Therefore, the measured results underestimate the power that would be obtained when the solar cells are implanted below the skin (up to ~20% under sun irradiation). In any case, the obtained power will primarily scale with the thickness of the skin.

Unlike skin, the optical filters have almost no scattering effect. The comparison of the calculated $I_{{{\text{SC}}\_{\text{C}}}}$, based on the transmission measurements and $I_{{{\text{SC}}\_{\text{M}}}}$, measured when the solar cells were covered with the different skin samples showed that scattering has an influence, since $I_{SC\_M}$ is larger than $I_{{{\text{SC}}\_{\text{C}}}}$. The optically opaque frame was larger than the solar cells, which enabled lateral irradiation by scattered light. In practice, the same (positive) effect would be present in a subcutaneously implanted solar cell. However, for a larger solar cell area (in this study 3.6 cm^2^), this effect will be small (<1%).

The non-ideal low light behavior of the solar cells leads to an additional slight underestimation of the maximum achievable energy output. In order to investigate the low light performance of the solar cells, voltage-current measurements were performed under irradiances that were reduced by up to three orders of magnitude as compared to full sunlight irradiation. It was found that for the used solar cells, $U_{\text{OC}}$ follows the log($I_{\text{SC}}$) in an ideal manner over a wide range of irradiance and therefore, the measured power output as shown in Fig. 6 is a reliable estimate of the maximum achievable power in the range above 10 *µ*W. In contrast, the solar cell’s performance deviates from ideal behavior in the 10 *µ*W range. Here, solar cells optimized for low light operation could harvest about 1.2 times more energy and more below.

### Study Data

In every season, the measured average power is multiple times higher than the power consumption of contemporary cardiac pacemakers (~5–10 *µ*W according to device manufacturer’s reference manuals). The participant with the lowest power still obtained 12 *µ*W, when considered the mean over every season.

Instead of measuring throughout a whole year including spring, it was decided to measure from summer to winter solstice. This period includes the sun’s full declination angle range and therefore the longest as well as the shortest day of the year. The most influential astronomical factor is the length of the day, therefore, the results may be applicable to the other half of the year. The mean power per month (Fig. 6) is continuously decreasing towards winter, which supports this assumption. When the days are shorter, less solar energy is available. This effect may even be intensified by the lower sun elevation, leading to more shadowing by high objects such as buildings or mountains. Additionally, people may spend less time outdoors when the weather is getting colder. This was also observed in the results, people spent 75% less days outdoors in winter compared to summer. In contrast, maximal power peaks throughout the day have the same amplitude in summer as well as in winter. The data was gathered in the northern hemisphere (Switzerland). Due to astronomical reasons the monthly tendency will be reciprocal in the southern hemisphere. The difference between summer and winter will be smaller towards the equator and higher towards the poles. The worldwide average global irradiation is higher than in Switzerland.

The average power was, as expected, higher when the weather was sunny (Fig. 7a). A relatively small difference is obtained between the weather situations cloudy and rainy, because in both situations, the sky is overcast. However, the generated power is not only dependent on the weather. In every season, significantly more power was generated when participants were outdoors (Fig. 7b). Overall, people spent more days indoors than outdoors (512 days indoors, 96 days outdoors). On one hand, the available irradiation indoor is given by the artificial illumination. Fluorescent lamps are often installed in offices, whereas at home more incandescent, halogen or LED lamps may be used. These light sources have different light spectra but all of them contribute to the overall power (Fig. 5c). On the other hand, weather also has an influence when subjects spend their time indoors. The available irradiation indoors is dramatically increased by solar radiation through the windows. As a consequence, the gained power indoors can vary from almost zero to more than 100 *µ*W.

Compared to the younger population, a large increase in prevalence rate of electronic implants such as pacemakers is obtained for the population of people 65 years or older, constantly increasing towards higher age^2^ and concurrently, the life expectancy of the worldwide population rises. Therefore, it is important to assess if there is an age-related trend in the measured power. Interestingly, people over 65 years obtained significantly more power. This may be explained by the fact that the elderly are retired and may have more leisure time which they can potentially spend outdoors if it is sunny. On the other hand, the younger population also obtained a multiple of the required power of a pacemaker. Thus, although they have a lower incidence rate for pacemaker implantation, a solar-powered implant would potentially save many re-interventions due to replacements throughout their life. For other electronic implants such as neural- or deep brain stimulators, the number of potentially saved re-interventions due to battery depletion is even higher, since the median age of patients receiving such an implant is lower and the longevity of these devices is shorter (2–7 years)^13^ compared to pacemakers. The higher power demand of these devices may be covered by an increased solar cell area or higher solar cell efficiencies in the future.

An active implant that is powered by solar cells has to feature an accumulator, that stores the surplus of the generated energy and ensures operation also during longer periods of darkness. The accumulator capacity has to be sufficient to power the implant for several months without any irradiation, especially for extreme cases as in very northern countries were the sun is not shining at all in some seasons. Furthermore, the accumulator has to fulfill special requirements concerning its cycle life. However, the accumulator capacity can be much lower and therefore smaller compared to implants powered by primary batteries. Furthermore, using ultrathin and flexible solar cells (e.g. CIGS cells,^4^ GaAs cells^10^) and flexible electronic circuitry dramatically reduces implant size, which may have an impact on complications related to the implantation procedure—besides the cosmetic aspect. The implant has to be hermetically covered by a translucent, inert and biocompatible material, which could be done by flexible multilayer packaging.^8^ Additionally, such an implant needs to have an alarm, warning the patient if the accumulator charge falls below a certain limit. In such cases, the implant could also be recharged with artificial light from outside.

To conclude, this publication reports the first real-life data of power generated by subcutaneous solar cells. With a few assumptions (e.g. solar cell area and efficiency, skin thickness/no skin), the results of this study can be scaled and applied to any other mobile, solar powered application on humans.

### Limitations

By advising the participants to cover the measurement device while the neck was covered, the reduction of power induced by clothes (e.g. scarf) was included in the results. However, shadowing of implanted solar cells by e.g. long hair could further negatively affect the power.

The optical filters mimic Caucasian skin type and therefore, the obtained power may be lower for darker skin types, which is mainly caused by the light absorption of melanin. However, the main absorption difference caused by melanin is obtained for ultraviolet light, which is also blocked by the filter, and decreases towards longer wavelengths.^18^ The transmittance curve emulated by the optical filters represents skin of 2.3 mm thickness. Although the typical skin thickness at the targeted implantation site (neck region) is smaller (epidermis + dermis = 1.4 ± 0.36 mm),^11^ the actual implantation depth may be higher which results in a decreased output power of the solar cells. In real life, subcoutaneously implanted solar cells will be operated at 10–15 °C higher temperature (~37 °C), which will lead to a power loss of about 1%.
